# Ultrasonic Microwave-Assisted Micelle Combined with Fungal Pretreatment of *Eucommia ulmoides* Leaves Significantly Improved the Extraction Efficiency of Total Flavonoids and Gutta-Percha

**DOI:** 10.3390/foods10102399

**Published:** 2021-10-10

**Authors:** Mingfang Wu, Peiyan Liu, Siying Wang, Chen Zhong, Xiuhua Zhao

**Affiliations:** 1 School of Biological and Chemical Engineering, Zhejiang University of Science and Technology, Hangzhou 310023, China; 2 College of Chemistry, Chemical Engineering and Resource Utilization, Northeast Forestry University, Harbin 150040, China; liupeiyan@nefu.edu.cn (P.L.); wsy0822@nefu.edu.cn (S.W.); 3 Key Laboratory of Forest Plant Ecology, Northeast Forestry University, Harbin 150040, China; 4 State Key Laboratory of Genetic Engineering, School of Life Sciences, Fudan University, Shanghai 200438, China; zhongchen@fudan.edu.cn

**Keywords:** *Eucommia ulmoides* leaves, ultrasonic microwave, flavonoids, gutta-percha, *Trichoderma viride*

## Abstract

A biological pretreatment of *Eucommia ulmoides* leaf biomass was carried out. Above all, the total flavonoids were isolated from *Eucommia ulmoides* leaves by the treatment of alkaline solution of sodium dodecyl benzene sulfonate combined with ultrasonic microwave. The extraction parameters were optimized by central composite design (CCD) software and were displayed as follows: surfactant concentration of 1.5%, microwave power of 700 W, extraction time of 30 min, and liquid–solid ratio of 50 mL/g. The actual yield of total flavonoids was 1.45%. The results of Sudan III color development showed that the cuticle of *Eucommia ulmoides* leaves was completely removed after dilute alkali pretreatment. Then, *Eucommia ulmoides* leaves were fermented by *Trichoderma viride* to remove the holocellulose and obtain gutta-percha. The content of cellulose and hemicellulose in *Eucommia ulmoides* leaves obviously decreased after *Trichoderma viride* fermentation. The optimum parameters were listed as follows: solid–liquid ratio of 0.06 g/mL, four extraction times, extraction time of 89.72 min, and extraction temperature of 85 °C. The actual yield of gutta-percha was 4.38%. The amount of extraction solvent per unit weight of gutta-percha from untreated *Eucommia ulmoides* leaves was 2.91 mL/mg, while that from leaves treated by *Trichoderma viride* was only 0.96 mL/mg. The amount of extraction solvent was grossly reduced, which is beneficial in terms of environmental protection. The characterizations of gutta-percha were verified by Fourier transform infrared spectroscopy (FTIR), X-ray diffraction (XRD), and proton nuclear magnetic resonance (^1^H NMR). This study laid a certain theoretical and experimental basis for the multi-stage extraction of *Eucommia ulmoides* leaves and the utilization of *Eucommia ulmoides* resources.

## 1. Introduction

**Figure 1:**
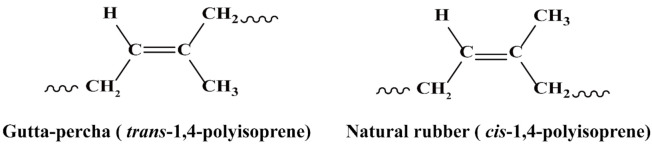
Macromolecular structure of gutta-percha and natural rubber.

*Eucommia ulmoides*, a member of *Eucommia ulmoides* family, is a large woody plant that mainly grows in China [1]. *Eucommia ulmoides* bark and leaves contain a variety of active components such as flavonoids, lignans, polysaccharides, and volatile oil. In particular, it harbors a special biomaterial, *Eucommia ulmoides* gum [2]. This gum is called balata or gutta-percha, a rubber with a molecular structure of *trans*-1,4-polyisoprene (Figure 1). The gutta-percha is an isomer of natural rubber (*cis*-1,4-polyisoprene) [3]. In addition to high flexibility (known as the general character of natural rubber), gutta-percha has also shown excellent plasticity. Moreover, gutta-percha possesses a number of unique properties such as high insulation, water resistance, acid and alkali resistance, and crystallization at room temperature [3,4]. Gutta-percha is an ideal material for the preparation of submarine cables, golf balls, and conveyor belts [5]. Unfortunately, *Eucommia ulmoides* tree needs to grow for more than 10 years to peel. Some improper maintenance after peeling is likely to cause diseases or insect pests, which is not conducive to the sustainable development of *Eucommia ulmoides* resources. Thus far, scholars have clarified that the active components of *Eucommia ulmoides* leaves are basically the same as those of *Eucommia ulmoides* bark [6]. *Eucommia ulmoides* is a deciduous tree that produces a large number of fallen leaves every year. Therefore, the development of effective components of *Eucommia ulmoides* leaves instead of *Eucommia ulmoides* bark is advantageous to the sustainable and healthy development of *Eucommia ulmoides* resources [7].

Flavonoids, one of the adequate and functional components in *Eucommia ulmoides*, has the activities of antioxidation, anti-tumor, anti-virus, and free radical scavenging [8,9]. The traditional extraction methods for flavonoids include Soxhlet extraction, heat reflux extraction, and alkaline water extraction [10,11]. However, they have multiple disadvantages such as long extraction time, high energy consumption, and low yield [12]. Ultrasonic microwave combined extraction technology is a new extraction technique for flavonoids [13,14]. This method makes full use of the cavitation effect of ultrasonic wave as well as the high energy and thermal effects of microwave extraction. It compensates the unequal defects of microwave heat and mass transfer produced by ultrasonic wave [15,16]. Compared with the traditional extraction method, the microwave ultrasonic extraction method has high safety and short time and can save energy (Table 1). Furthermore, the excellent thermal effect of microwave can effectively make up for the insufficient heat production by ultrasound [17]. The rapid and efficient extraction of samples under low temperature and atmospheric pressure was realized [18]. However, these extraction methods usually use traditional organic solvents such as chloroform, methanol, ethanol, acetone, and ethyl acetate as extraction media [19]. The use of organic solvents has numerous disadvantages, including strong volatility, high toxicity, and serious pollution [20]. Surfactant has a special amphiphilic structure that can form molecular liquid membrane extraction, increase the liquid–solid contact area, and enhance the wettability and permeability of the solvent to the material [21]. Microwave heating can destroy the cell wall of plants and improve the porous permeability and water absorption of tissue cells [22,23]. The combination of ultrasonic-microwave extraction and surfactant is able to decrease the cost and improve the extraction efficiency of flavonoids [24,25]. At present, the extraction method of total flavonoids from *Eucommia ulmoides* leaves by ultrasonic microwave combined with surfactant has not been reported.

Yan et al. demonstrated that the content of gutta-percha in *Eucommia ulmoides* leaves was 1.55–3.23% [26]. Generally, the gutta-percha is extracted with traditional organic solvent petroleum ether, but the extraction rate is low [1]. In terms of the enhancement of the yield of gutta-percha, the pretreatment of fermentation technique is an important step in the early stage of gutta-percha extraction. *Trichoderma viride* is an aerobic heterotrophic fungus and one of the strains with the highest cellulase activity [27]. *Trichoderma viride* has the characteristics of low cost and high enzyme production. The production of cellulase by solid-state fermentation of *Trichoderma viride* has become the technical development direction in cellulase industrial production [28]. However, few reports are available to guide the gutta-percha isolation from *Eucommia ulmoides* leaves by *Trichoderma viride* fermentation. Pretreatment of plant tissue by *Trichoderma viride* fermentation can degrade cellulose, pectin, and lignin in the plant cell wall into monosaccharides and oligosaccharides [29]. Thus, the dense structure of the cell wall is destroyed, and the effective components in the cell are easy to flow out [30]. The pretreatment is not only easy to operate, but it does achieve high yield for the extraction of gutta-percha with turpentine. The purpose of this study was to efficiently extract total flavonoids and gutta-percha from *Eucommia ulmoides* leaves by using environmentally friendly solvent.

**Figure 2:**
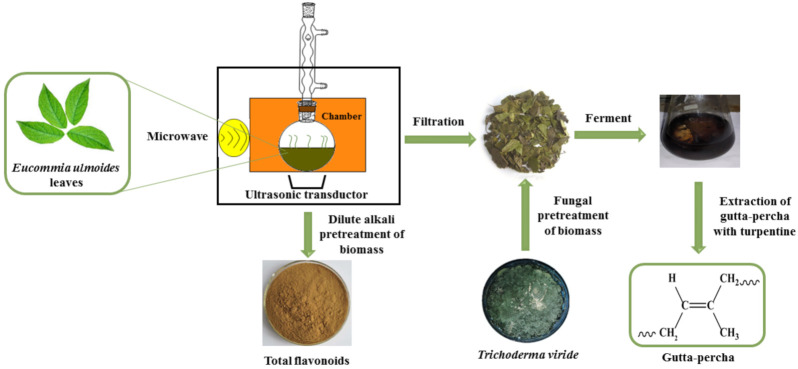
A schematic diagram of the extraction process.

In the first part of this study, the extract parameters of total flavonoids were optimized. A schematic diagram of the extraction process is shown in Figure 2. The content of surfactant, the ratio of liquid to solid, microwave power, and extraction time were investigated to obtain the optimum extraction conditions. In the second part, the combination of *Trichoderma viride* pretreatment and turpentine extraction was used to obtain a higher yield of gutta-percha. In addition, XRD, FTIR, and ^1^H NMR were used to characterize gutta-percha.

## 2. Materials and Methods

### 2.1. Materials

Fresh *Eucommia ulmoides* leaves were purchased from Bozhou Chinese herb market (Anhui, China). The *Eucommia ulmoides* leaves were dried in a cool and ventilated place at room temperature for 20 days, and then stored in a dry place until use.

Sodium dodecyl benzene sulfonate (SDBS, analytical purity) was acquired from Wuxi Yatai United Chemical Co., Ltd. (Wuxi, Jiangsu, China). *Trichoderma viride* (serial number: Bio-52626) was purchased from Beijing Biobw Biotechnology Co., Ltd. (Beijing, China). Turpentine (analytical purity) and petroleum ether (analytical purity) were acquired from Tianjin Fuyu Fine Chemical Reagent Co., Ltd. (Tianjin, China).

### 2.2. Dilute Alkali Pretreatment of Biomass

The *Eucommia ulmoides* leaves were pretreated with alkaline sodium dodecyl benzene sulfonate solution, which is beneficial to the extraction of total flavonoids. Microwave ultrasonic extraction combined with alkaline SDBS solution was used to extract total flavonoids from *Eucommia ulmoides* leaves. In brief, 6 g of the dried *Eucommia ulmoides* leaves was mixed with 300 mL of the alkaline SDBS solution in round-bottomed flasks so that the *Eucommia ulmoides* leaves were completely immersed in the extraction solution and soaked at room temperature for 6 h. Subsequently, the mixture system was placed in a microwave ultrasonic extraction device for further extraction. Fixed ultrasonic power was 50 W for a subsequent one-variate optimization experimental design. Four factors affecting the yield of total flavonoids in the extraction process were investigated by single-factor experiment: content of surfactant SDBS (0, 0.5, 1, 1.5, and 2%), liquid–solid ratios (20, 40, 60, 80, and 100 g/mL), microwave power (100, 300, 500, 700, and 900 W), and extraction time (10, 20, 40, 60, and 80 min). The filtrate and the filter residue were separated by filtration post-extraction. Sodium nitrite–aluminum nitrate method combined with ultraviolet spectrophotometry was used to detect the total flavonoid content of a 1 mL extract. The residue was then dried for the next step (gutta-percha extraction).

### 2.3. Process Optimization

Interactions between each factor are crucial for obtaining higher yield of total flavonoids. After selecting the most important factors for total flavonoid extraction, we investigated the influence of operating parameters through a central composite design (CCD) of RSM using Design Expert 8.0.6.1 software. On the basis of CCD, we optimized the effects of surfactant SDBS content, liquid–solid ratio, microwave power, and extraction time on the extraction rate of total flavonoids from *Eucommia ulmoides* leaves. The scope of the variables studied are listed as follows: 1–2% content of surfactant SDBS, 40–60 g/mL liquid–solid ratios, 500–900 W microwave power, and 2040 min extraction time. Prediction of the response through the full second-order polynomial equation is as shown in Equation (1):

(1) $$y=\beta_{0}+\overset{k}{\underset{i=1}{\sum }}\beta_{i}x_{i}+\overset{k}{\underset{i=1}{\sum }}\beta_{ii}x_{i}{}^{2}+\overset{k}{\underset{i<j}{\sum }}\beta_{ij}x_{i}x_{j}$$

In the above formula, *y* is the predicted response value, *β*_0_ is the coefficient constant, *β_i_* is the linear coefficient, *β_ii_* is the quadratic equation coefficient, and *β_ij_* is the interaction coefficient. Four different independent variables are defined as *X*_1_, *X*_2_, *X*_3_, and *X*_4_.

### 2.4. Chromogenic Experiment of Horny Layer

Sudan III dye has a good dyeing effect on the horny layer of plant leaves. The change of staining was used as the basis to judge whether the horny layer of *Eucommia ulmoides* leaves was removed completely after pretreatment with dilute alkali solution. We prepared 0.1% Sudan III solution by using 95% ethanol solution as solvent. The original leaves of untreated *Eucommia ulmoides* and the leaves of *Eucommia ulmoides* treated with dilute alkali were fully soaked in water. Next, the above samples were immersed in Sudan III solution for 5 min. The excess liquid from the blade was absorbed with filter paper and observed with the optical microscope.

### 2.5. Removal of Holocellulose by Trichoderma viride Fermentation

The leaf residue of *Eucommia ulmoides* after dilute alkali treatment was collected and further fermented with *Trichoderma viride* to remove holocellulose.

#### 2.5.1. Optimization of Fermentation Conditions of *Trichoderma viride*

*Trichoderma viride* was inoculated into the test tube containing potato dextrose agar (PDA) medium and cultured at 28 °C for 7 days. A ring of pollution-free green hyphae was inoculated in Fernbach flask containing 100 mL seed medium (CMC-Na: 7.5 g/L, peptone: 5 g/L, tween-80: 2 mL/L, MgSO_4_·7H_2_O: 0.3 g/L, CaCl_2_: 0.3 g/L, FeSO_4_·7H_2_O: 0.005 g/L, MnSO_4_·H_2_O: 0.0016 g/L, ZnSO_4_·7H_2_O: 0.0014 g/L, and CoCl_2_: 0.002 g/L). The culture bottle was cultured in the incubator at a constant temperature of 28 °C and 150 r/min for 72 h.

Biodegradation of *Eucommia ulmoides* leaves by *Trichoderma viride* is closely related to inoculum size, solid–liquid ratio, pH, and culture time. The pH value of synthetic medium (NaCl: 0.5g/L, MgSO_4_: 0.5 g/L, (NH_4_)2SO_4_: 13 g/L, KH_2_PO_4_: 10.5 g/L, CaCl_2_: 0.3 g/L, FeSO_4_·7H_2_O: 0.01 g/L, MnSO_4_·7H_2_O: 0.015 g/L, ZnSO_4_·7H_2_O: 0.015 g/L, and CoCl_2_: 0.01 g/L) was adjusted by citric acid buffer solution (pH = 4.8). The *Eucommia ulmoides* leaves treated in Section 2.2 and the synthetic culture medium were fully mixed in the Fernbach flask for 6 h according to a certain ratio of solid to liquid. Next, the culture medium system was put into a high-pressure steam sterilizer and sterilized at 120 °C for 15 min. When its temperature dropped to room temperature, a certain volume ratio of *Trichoderma viride* suspension was added under aseptic conditions. It was fermented for 8 days at 28 °C and 120 r/min in an oscillatory incubator. After fermentation, the suspension 0.2 mL was absorbed, and the enzyme activity was determined by DNS method.

In this study, the orthogonal experimental design was used to determine the optimum technological conditions. The inoculum size ranged from 20 to 30%. The ratios of solid-to-liquid were within 1:10–1:50. The pH was between 4 and 6, and the culture time ranged from 3 to 7 days. The results are shown in Table 1.

#### 2.5.2. Determination of Enzyme Activity

The fermentation medium of 0.2 mL *Trichoderma viride* was accurately absorbed and put into the test tube, in which CMC-Na (0.1%, 0.9 mL) and HAc-NaAc (pH 5.0, 0.9 mL) were added and saccharified in a 55 °C water bath for 30 min [31]. Subsequently, the 2 mL added DNS reagent was mixed and heated in boiling water bath for 5 min, and the final temperature was reduced to room temperature. Distilled water was added to a constant volume (25 mL), and its absorbance value was determined at 540 nm. The standard curve of glucose is as follows: *y* = 0.9229*x* − 0.006 (*R*^2^ = 0.9994). The formula (Equation (2)) for calculating unit enzyme activity is as follows:

(2) $$\mathrm{Enzyme}\;\mathrm{activity}\;\left(U/\mathrm{mL}\right)=\frac{OD\times \frac{1}{K}\times n\times 1000}{T}$$

*OD*: absorbance of the enzyme solution; *K*: slope of the curve; *n*: dilution multiple; *T*: reaction time (min).

#### 2.5.3. Method for Determination of Lignin, Cellulose, and Hemicellulose

The content of lignin in the sample was determined by Klason method [32]. In short, the sample and the mixture of ethanol and benzene were extracted for 6 h by Soxhlet extractor. A total of 1 g dried sample was placed into a known constant weight crucible (*W*_3_), and the whole system was burned in a 600 °C high temperature furnace. The weighing mass of the system was *W*_2_ after cooling (Equation (3)).

(3) $$W_{0}\left(\%\right)=\frac{W_{2}-W_{3}}{W_{1}}\text{×}\;100$$

*W*_0_: ash content of *Eucommia ulmoides* leaves (%); *W*_2_: ash and crucible total mass (g); *W*_3_: crucible mass (g); *W*_1_: total mass of sample (g).

A total of 1 g dried sample after extraction and 15 mL 72% concentrated sulfuric acid were added to react at room temperature for 4 h. Next, water was added to the above system until the concentration of sulfuric acid became 3%, and then the whole system was boiled and refluxed for 4 h. Finally, a large number of insoluble residues were obtained by filtration, and the mass was named as *W*_4_ after drying at 105 °C (Equation (4)).

(4) $$\mathrm{Lignin}\;\mathrm{content}=\frac{W_{4}}{W_{5}}\times 100-W_{0}$$

*W*_4_: product quality after acid hydrolysis (g); *W*_0_: ash content of *Eucommia ulmoides* leaves (%); *W*_5_: total mass of sample (g).

The content of cellulose in *Eucommia ulmoides* leaves was determined by anthrone reagent colorimetry [33]. In brief, the dried *Eucommia ulmoides* leaves samples were put into a 100 mg volumetric flask and digested with 60 mL 60% sulfuric acid solution for 30 min. The volume is then fixed to the calibration line with 60% sulfuric acid solution. The above solution was absorbed for 5 mL and placed in a 100 mL volumetric bottle, and the volume was fixed with ultra-pure water to the calibration line. The absorbance of the above solution was measured by adding 0.5 mL 2% anthrone reagent and 5 mL concentrated sulfuric acid for 10 min at 620 nm wavelength. The formula of cellulose standard curve is as follows: *y* = 0.0082*x* + 0.0593 and *R*^2^ = 0.9997 (where *x* is cellulose concentration (mg/mL) and *y* is the peak area) Equation (5).

(5) $$Y=\frac{X\times 10^{-6}\times A\times 100}{W}$$

*Y*: cellulose content in the sample (%); *X*: according to the regression equation to calculate the cellulose content (μg); *A*: multiple of dilution of the sample; *W*: sample weight (g).

The content of hemicellulose in *Eucommia ulmoides* leaves was determined by 2 mol/L hydrochloric acid hydrolysis method, and the content of reducing sugar was determined by DNS method [34]. The sample of 0.1 g *Eucommia ulmoides* leaves was added 10 mL 80% Ca(NO_3_)_2_ solution to boil for 5 min and then centrifuged to obtain precipitation. Then, 10 mL 2 mol/L Hydrochloric acid solution was added to the sediment in boiling water bath for 45 min. The suspension was centrifuged, and the supernatant was neutralized by NaOH titration. A total of 2 mL of the above solution was added with 1.5 mL DNS reagent, and then the absorbance was determined at 540 nm after reacting for 5 min in boiling water bath. The equation of glucose standard curve is as follows: *y* = 0.9229*x* − 0.005 and *R*^2^ = 0.9994 (where *x* is glucose concentration (mg/mL) and *y* is the peak area). The content of reducing sugar was multiplied by 0.9 to obtain the content of hemicellulose.

### 2.6. Extraction and Purification of Gutta-Percha

The raw material of the extraction comes from the dried *Eucommia ulmoides* leaves after the pretreatment of *Trichoderma viride*. Gutta-percha was extracted from *Eucommia ulmoides* leaves by using DF–101S collector constant temperature heating magnetic agitator (Yuhua, Shanghai, China). The temperature in the extraction process was controlled by the temperature sensor. The dried *Eucommia ulmoides* leaves were accurately weighed and put into the round bottom flask according to a certain proportion with turpentine to extract gutta-percha at a constant stirring speed. The ratio of solid to liquid (0.03, 0.05, 0.07, 0.1, and 0.2 g/mL), extraction times (1, 2, 3, 4, and 5 times), extraction time (30, 60, 90, 120, and 150 min), and extraction temperature (55, 65, 75, 85, and 95 °C) were investigated by single-factor experiment. On the basis of CCD, we optimized the effects of solid-liquid ratio, extraction times, extraction time, and extraction temperature on the extraction rate of gutta-percha from *Eucommia ulmoides* leaves. The scope of the variables studied were ratio of solid to liquid of 0.03–0.1 g/mL, extraction times of 1–5 times, extraction time of 30–150 min, and extraction temperature of 55–95 °C.

After the extraction, the insoluble residue was filtered to obtain the extract. When the extract was cooled to room temperature, twofold volume of anhydrous ethanol was added to it, and the white solid appeared immediately. The gutta-percha in the extract was fully precipitated by refrigeration in the freezer at −20 °C. The suspension was centrifuged at 10,000 r/min for 10 min and dried in the oven at 60 °C to obtain white gutta-percha. The formula (Equation (6)) for calculating the yield of gutta-percha is as follows:

(6) $$\eta =\frac{W_{1}}{W_{0}}\times 100\%$$

η: yield of gutta-percha (%); *W*_1_: mass of gutta-percha (g); *W*_0_: total mass (g) of *Eucommia ulmoides* leaves.

### 2.7. Effect of Trichoderma viride Pretreatment on the Yield of Gutta-Percha

The leaves of *Eucommia ulmoides* were compared with those fermented by *Trichoderma viride*. *Eucommia ulmoides* leaves (untreated) and *Eucommia ulmoides* leaves fermented by *Trichoderma viride* were used as extraction materials (5 g). We added 100 mL turpentine and extract at 85 °C for 60 min, and then cooled the mixture to room temperature. The insoluble residue was removed, and the crude gum was obtained by adding ethanol. Gutta-percha was obtained after storage for the night at −20°C, centrifugation with 10,000 r/min for 10 min, and drying at 60 °C.

### 2.8. Scanning Electron Microscope (SEM)

The morphology of *Eucommia ulmoides* leaves was detected by SEM (Quanta 200-FEI Company, Eindhoven, The Netherlands). Coating the sample evenly on the gold-plated conductive adhesive (JFC 1200 Fine Coating Machine, JEOL, Japan).

### 2.9. X-ray Diffraction (XRD)

The crystal forms of *Eucommia ulmoides* leaves and gutta-percha were detected and analyzed by X-ray diffractometer (Philips, Amsterdam, the Netherlands). The samples were irradiated with copper target tube and detected under the condition of 40 kV, 30 mA, 10° < 2θ < 80°.

### 2.10. Fourier Transform Infrared (FTIR)

The solid properties of gutta-percha were detected by FTIR spectrometer. The measured frequency range was 4000 to 400 cm^−1^, and the resolution was 1 cm^−1^.

### 2.11. ^1^H NMR Analysis

Accurate weighing of 20 mg gutta-percha dissolved in TOLUENE-D8 containing tetramethylsilane as an internal standard with a chemical shift of δH = 0 was performed. The scope of the ^1^H NMR spectrum was from 0 to 8 ppm. All the ^1^H NMR spectra were acquired on a Bruker 500MHz spectrometer (Bruker BioSpin AG, Fällanden, Switzerland).

## 3. Results and Discussion

### 3.1. Optimization of Biomass Pretreatment Process for Eucommia ulmoides Leaves

#### 3.1.1. Effects of the Surfactant

**Figure 3:**
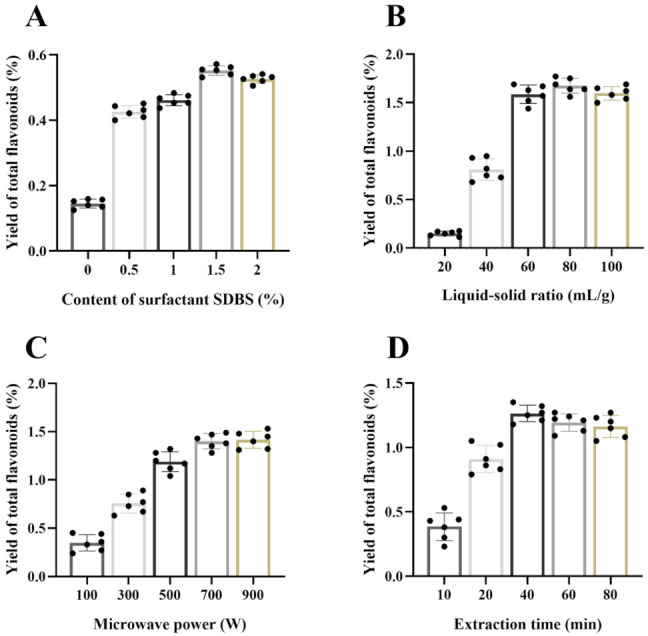
Optimization of extraction total flavonoids method by single-factor design (data shown as means ± SD, *n* = 6). Effect of surfactant content (**A**), liquid–solid ratio (**B**), microwave power (**C**), and extraction time (**D**).

Different content of surfactant SDBS (0, 0.5, 1, 1.5 and 2%) combined with fixed conditions (500 W microwave power, 90 °C extraction temperature, 20 mL/g liquid–solid ratio, 10 min extraction time) was used to extract the sample. As shown in Figure 3A, the results showed that with the increase of the content of surfactant SDBS from 0% to 1.5%, the yield of total flavonoids increased gradually (0.156 ± 0.006, 0.442 ± 0.053, 0.473 ± 0.062, and 0.556 ± 0.052%). However, with the increase of surface-active dose to 2%, the yield of total flavonoids did not change significantly (0.526 ± 0.046%). The increase of surfactant content could increase the solubility of total flavonoids in the system solution. However, with the continuous increase of surfactant content, the yield of total flavonoids reached the maximum and then maintained balance, which may have been due to the limited content of total flavonoids.

#### 3.1.2. Effect of the Liquid–Solid Ratio

The effect of the ratio of liquid to solid on the yield of flavonoids was verified. Under the conditions of different liquid–solid ratios (20, 40, 60, 80, and 100 mL/g), the sample was extracted with 1% surfactant SDBS, 500 W microwave power, 90 °C extraction temperature, and 10 min extraction time. The results (Figure 3B) displayed that the yield of total flavonoids increased with the increase of liquid–solid ratio from 20 mL/g to 60 mL/g (0.15 ± 0.012, 0.82 ± 0.052, and 1.63 ± 0.091%). However, increasing the ratio of liquid to solid (80 mL/g and 100 mL/g) could not increase the yield of total flavonoids (1.69 ± 0.084 and 1.62 ± 0.082%, respectively). Once the amount of solvent is not enough, it will often lead to incomplete extraction, but excessive solvent may lead to unnecessary waste [35]. Therefore, to save solvent dosage, we selected the range of liquid–solid ratio from 60 mL/g to 80 mL/g in the following experiments.

#### 3.1.3. Effect of the Microwave Power and Extraction Time

Under the conditions of different microwave power (100, 300, 500, 700, and 900 W), we extracted the sample with 1% surfactant SDBS, 60 mL/g liquid–solid ratio, 90 °C extraction temperature, and 10 min extraction time. With the increase of microwave power, the yield of total flavonoids was increased (Figure 3C). When the microwave power increased from 700 W to 900 W, the yield of total flavonoids did not increase significantly. Thus, the microwave power was controlled in the range of 700 to 900 W. The internal water molecules in the cell absorbed the microwave to increase the internal temperature rapidly. The pressure generated by liquid water vaporization in the cell broke through the cell membrane and cell walls, forming small cavities. The micelle molecules can carry flavonoids and lipid-soluble components out of the extracellular solvent medium, thus obtaining high concentrations of target products.

Under the conditions of different extraction times (10, 20, 40, 60 and 80 min), we extracted the sample with 1% surfactant SDBS, 500 W microwave power, 90 °C extraction temperature, and 60 mL/g liquid–solid ratio. When the extraction time increased from 10 min to 40 min, the yield of total flavonoids was increased (Figure 3D). The results demonstrated that the increase of system temperature was beneficial to the dissolution of flavonoids. However, a long-time extraction was not conducive to the stability of flavonoids’ structures, which may result in a decrease in yield.

### 3.2. Optimization of Extraction Technology of Total Flavonoids by CCD Software

The interactions of various factors (the content of surfactant SDBS: *X*_1_, microwave power: *X*_2_, extraction time: *X*_3_, and liquid–solid ratio: *X*_4_) in the quadratic polynomial model for the extraction of total flavonoids was further explored (Table 2). As shown in Table 3, the “lack of fit *p*-value” of 0.2039 > 0.05 revealed that quadratic regression model fitted with the actual situation. The *R*^2^ value of the equation was 0.9852, and the correlation coefficient was closer to 1, indicating that 98.52% of the experimental data can be explained by the model. The correction determination coefficient *R_adj_*^2^ was 0.9761, suggesting that a good linear correlation between independent variables. The quadratic polynomial equation of the correlation between the response variables and the test variables is as follows:

*Y* = 1.64 + 0.18*X*_1_ − 0.090*X*_2_ + 0.077*X*_3_ + 0.22*X*_4_ + 0.033*X*_1_*X*_2_ − 0.041*X*_2_*X*_4_ − 0.044*X*_3_*X*_4_ − 0.12*X*_1_^2^ − 0.15*X*_2_^2^ − 0.023*X*_3_^2^ − 0.032*X*_4_^2^

**Figure 4:**
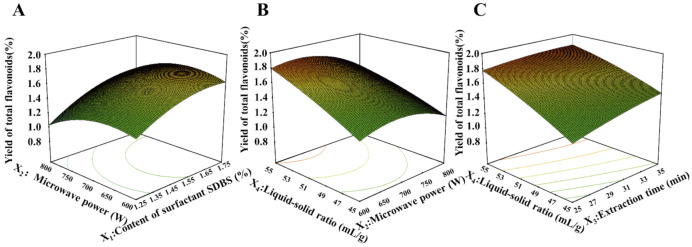
Response surface for the interactions of independent variables on extraction efficiency of total flavonoids. (**A**) The interaction of surfactant content and microwave power, (**B**) the interaction of microwave power and liquid–solid ratio, and (**C**) the interaction of extraction time and liquid–solid ratio.

A 3D surface chart was obtained according to the above equation (Figure 4A). The effects of surfactant SDBS content (*X*_1_) and microwave power (*X*_2_) are shown by 3D surface (Figure 4A). The yield of total flavonoids increased with the increase of surfactant concentration. When the microwave power increased from 600 to 700W, the yield increased gradually, and decreased with the increase of microwave power. Figure 4B shows the 3D surface chart of microwave power (*X*_2_) and liquid–solid ratio (*X*_4_). With the increase of liquid–solid ratio from 45 mL/g to 55 mL/g, the yield of total flavonoids clearly increased. The yield increased while the microwave power changed from 600 to 700W and decreased slightly with increased microwave power. Figure 4C shows a 3D surface chart view of the extraction time (*X*_3_) and liquid–solid ratio (*X*_4_). The yield of total flavonoids increased obviously with the increase of liquid–solid ratio. Meanwhile, the yield of total flavonoids increased with the increase of extraction time from 25 to 35 min. It was shown from the three 3D surface charts that the concentration of surfactant had a greater effect on the yield of total flavonoids. The optimum conditions predicted by response surface software were as follows: 1.5% surfactant concentration, 700 W microwave power, 30 min extraction time, and 50 mL/g liquid–solid ratio. Under the conditions of the point prediction, the yield of total flavonoids was 1.45%.

### 3.3. Characterization of Removal Effect of Stratum Corneum

**Figure 5:**
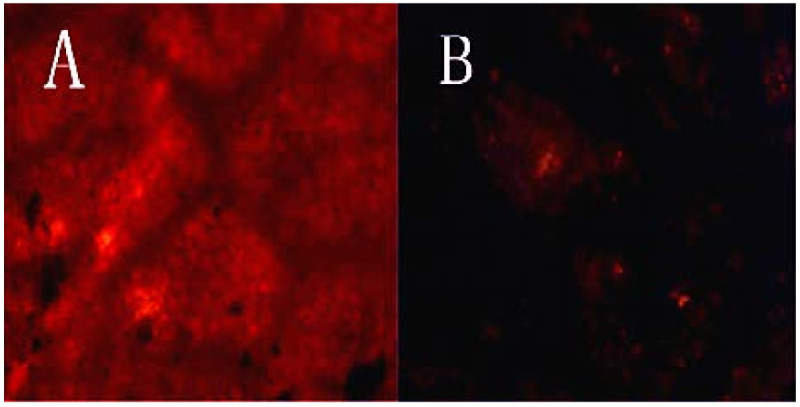
Sudan III staining on the surface of *Eucommia ulmoides* leaves observed under optical microscope (10 × 10 magnification). (**A**) Leaves of *Eucommia ulmoides* without pretreatment; (**B**) leaves of *Eucommia ulmoides* after dilute alkali pretreatment.

The leaves of *Eucommia ulmoides* were characterized with Sudan III dye. As shown in Figure 5A, the leaves of *Eucommia ulmoides* without pretreatment showed a large red area after staining with Sudan III. The results showed that the surface of *Eucommia ulmoides* leaves was covered with a horny layer. The stratum corneum hindered the acquisition of biomass and was not conducive to the extraction of gutta-percha by *Trichoderma viride* fermentation. The results of Sudan III staining of *Eucommia ulmoides* leaves pretreated with dilute alkali solution are shown in Figure 5B. The red area of *Eucommia ulmoides* leaves disappeared, indicating that the removal of stratum corneum is relatively complete.

### 3.4. Optimization of Fermentation Conditions of Trichoderma viride

The biodegradation of *Eucommia ulmoides* leaves by *Trichoderma viride* was closely related to the yield of gutta-percha. In this study, the optimum process conditions were determined by orthogonal experimental design method. In the orthogonal experimental design, the optimum fermentation technology of *Trichoderma viride* was determined by nine orthogonal experiments (L_9_3^4^) with inoculum amount, solid–liquid ratio, pH value, and culture time as the factors. Table 4 shows the orthogonal experimental results of enzyme activity between 2.348 and 19.882. A thorough analysis of the tabular data showed the enzyme activity of the maximum factor for the optimal conditions. The optimum fermentation conditions of *Trichoderma viride* were as follows: the pH value was 6, the ratio of solid to liquid was 1 to 10, the amount of inoculum was 20%, and the culture time was 5 days.

### 3.5. Content of Lignin, Cellulose, and Hemicellulose

**Figure 6:**
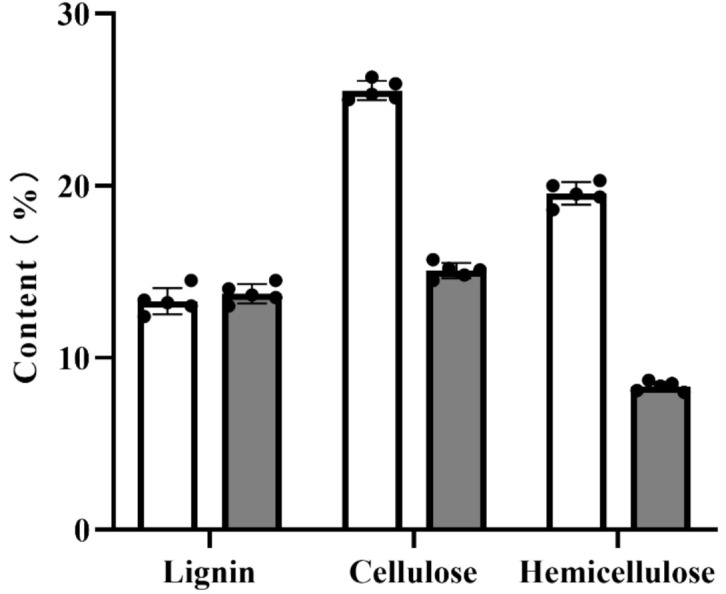
Contents of lignin, cellulose, and hemicellulose in *Eucommia ulmoides* leaves (data shown as means ± SD, *n* = 5).

The contents of lignin, cellulose, and hemicellulose in *Eucommia ulmoides* leaves without any treatment were 13.35%, 25.93%, and 19.33%, respectively (Figure 6). The contents of lignin, cellulose, and hemicellulose in *Eucommia ulmoides* leaves after fermentation by *Trichoderma viride* were 13.64%, 14.81%, and 8.35%, respectively. After the *Eucommia ulmoides* leaves were fermented by *Trichoderma viride*, the lignin did not clearly change, but the contents of cellulose and hemicellulose decreased remarkably. The content of lignin in *Eucommia ulmoides* leaves fermented by *Trichoderma viride* did not change significantly, but the contents of cellulose and hemicellulose decreased significantly. Because *Trichoderma viride* had a good degradation effect on the amorphous region of cellulose, the fermentation treatment of *Trichoderma viride* had a high removal effect on the cellulose in *Eucommia ulmoides* leaves. In addition, the content of hemicellulose also decreased. This was due to the fact that *Eucommia ulmoides* leaves promote the hydrolysis of hemicellulose under the action of ultrasound in dilute alkali solution, which leads to the decrease of hemicellulose content in *Eucommia ulmoides* leaves.

### 3.6. Optimization of Extraction Conditions of Gutta-Percha

The raw material was *Eucommia ulmoides* leaves pretreated by *Trichoderma viride* fermentation, and the extraction solvent was turpentine; gutta-percha was extracted by constant temperature stirring. The optimum extraction conditions were determined by single-factor experiment and CCD software.

#### 3.6.1. Effect of the Solid–Liquid Ratio

**Figure 7:**
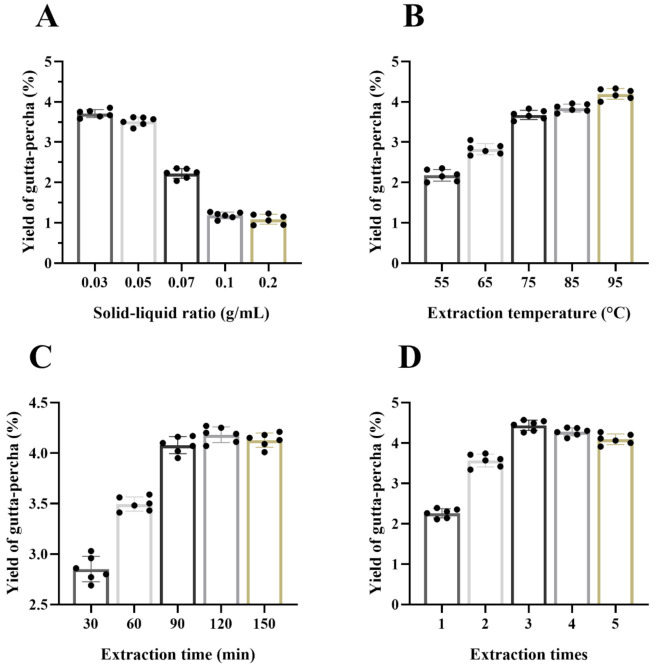
Optimization of extraction gutta-percha method by single-factor design (data shown as means ± SD, *n* = 6). Effect of solid–liquid ratio (**A**), extraction temperature (**B**), extraction time (**C**), and extraction times (**D**).

The samples were extracted at 80 °C and different solid–liquid ratios (0.03, 0.05, 0.07, 0.1, and 0.2 g/mL). The extraction time was 60 min, and the extraction times was two times. Figure 7A shows that the yield of gutta-percha decreased with the increase of solid–liquid ratio. This phenomenon shows that insufficient extraction solvent will lead to incomplete extraction. Therefore, the follow-up optimization experiment was carried out by controlling the solid–liquid ratio in the range of 0.03–0.1 g/mL.

#### 3.6.2. Effect of the Extraction Temperature

Under different extraction temperatures (55, 65, 75, 85, and 95 °C), the sample of gutta-percha was obtained by heating for 60 min with a 0.03 g/mL solid–liquid ratio for two times. When the temperature increased from 55 °C to 95 °C, the yield of gutta-percha increased significantly (Figure 7B). Increasing the extraction temperature will decrease the solvent viscosity and increase the solvent permeability. The penetration of turpentine into the sample powder is promoted by irregular molecular motion [36]. When the temperature was above 95 °C, the extraction rate of gutta-percha was not improved. Therefore, the reaction temperature range from 55 to 95 °C was selected for follow-up study.

#### 3.6.3. Effect of the Extraction Time

Under the conditions of 85 °C reaction temperature, 0.03 g/mL solid–liquid ratio, two extraction times, and different extraction times (30, 60, 90, 120 and 150 min), we determined the optimum extraction time of gutta-percha from turpentine. Figure 7C shows that the yield of gutta-percha increased significantly with the increase of extraction time from 30 to 120 min. However, the yield did not increase with the increase of extraction time from 120 to 150 min. The fact that the extraction time of 120–150min did not increase the yield of gutta-percha may have been due to the maximum solubility of the extraction solvent. Therefore, to avoid the decomposition of effective substances caused by long-term heating, we selected the heating time of 30–120 min for the follow-up experiment.

#### 3.6.4. Effect of the Extraction Times

Turpentine was used as extraction solvent to extract gutta-percha from *Eucommia ulmoides* leaves. The effects of different extraction times (1, 2, 3, 4, and 5) on the yield of gutta-percha were investigated under the conditions of 85 °C reaction temperature, 0.03 g/mL solid–liquid ratio, and 60 min extraction time. With the increase of extraction times, the yield of gutta-percha increased (Figure 7D). With the increase of extraction times from three times to five times, the yield of gutta-percha did not increase. Thus, extraction times of 2–4 were selected for the subsequent experiments.

#### 3.6.5. Optimization of Extraction Technology of Gutta-Percha by CCD Software

In this study, taking the yield of gutta-percha as the response value, we fitted the interactions among solid–liquid ratio (*X*_1_, g/mL), extraction times (*X*_2_), extraction time (*X*_3_, min), and extraction temperature (*X*_4_, °C) (Table 5). The level of factors is listed in Table 6. The multivariate quadratic regression equation model was obtained as follows:

*Y* = 3.78 − 0.38*X*_1_ + 0.26*X*_2_ − 0.00591*X*_3_ + 0.16*X*_4_ + 0.26*X*_1_*X*_4_ + 0.34*X*_2_*X*_4_ − 0.47*X*_1_^2^ − 0.33 *X*_2_^2^ − 0.32*X*_3_^2^ − 0.014*X*_4_^2^

**Figure 8:**
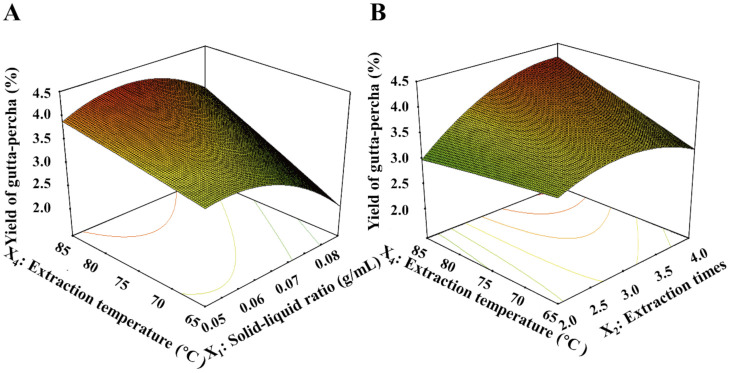
Response surface for the interactions of independent variables on extraction efficiency of gutta-percha. (**A**) The interaction of solid–liquid ratio and extraction temperature; (**B**) the interaction of extraction times and extraction temperature.

The “lack of fit *p-*value” of 0.7129 > 0.05 showed that the quadratic regression model fitted well with the actual situation. The *R*^2^ value of the equation was 0.8897, which shows that the model has a high degree of fitting with the predicted data, and therefore it was proven that the model is highly significant. The 3D surface chart can directly reflect the influence of various factors and their interaction on the response value (Figure 8). Figure 8A exhibits the 3D surface chart of solid–liquid ratio (*X*_1_) and extraction temperature (*X*_4_). The yield of gutta-percha increased significantly with the increase of extraction temperature from 65 to 85 °C. The yield increased significantly when the ratio of solid to liquid increased from 0.05 to 0.07 and decreased slightly with the increase of the ratio of solid to liquid. Figure 8B shows the 3D surface diagram of extraction time (*X*_2_) and extraction temperature (*X*_4_). The yield of gutta-percha increased significantly with the increase of extraction temperature. When the extraction times increased from two times to three times, the yield of gutta-percha increased significantly, and the yield tended to be stable with the extension of extraction times. It was shown from the two 3D surfaces chart that the solid–liquid ratio had a greater effect on the yield of gutta-percha. The optimum conditions predicted by response surface software were 0.06 g/mL solid–liquid ratio, four extraction times, 89.72 min extraction time, and 85 °C extraction temperature. In addition, the further results demonstrated that the yield of gutta-percha reached 4.38% on the basis of the point prediction-guided conditions.

#### 3.6.6. Comparison of Yield of Gutta-Percha before and after Pretreatment

In this study, turpentine was used as extraction solvent to contrast the yield of gutta-percha between untreated *Eucommia ulmoides* leaves and *Trichoderma viride* treatment. The results show that the gutta-percha obtained from *Eucommia ulmoides* leaves treated with *Trichoderma viride* (2.77%) was significantly improved, as shown in Table 7. This was because the *Eucommia ulmoides* leaves fermented by *Trichoderma viride* can destroy the fiber structure of *Eucommia ulmoides* leaves so that the fiber structure of *Eucommia ulmoides* leaves become loose. Porousness is conducive to the penetration of solvents; thus, the extraction efficiency was increased. Through the calculation, we can conclude that the amount of solvent needed for the extraction of gutta-percha per unit mass is 2.91 mL/mg (untreated) and 0.96 mL/mg (treated). The *Eucommia ulmoides* leaves treated by *Trichoderma viride* reduced the amount of solvent, thus reducing the environmental pollution.

### 3.7. SEM Analysis

**Figure 9:**
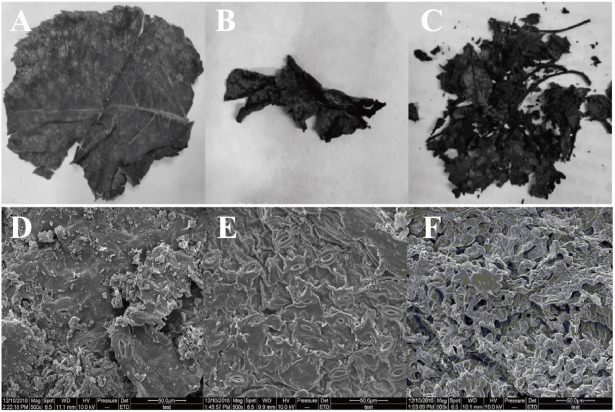
The morphology of the sample under scanning electron microscope. Image of *Eucommia ulmoides* leaves: (**A**) dried raw leaves, (**B**) leaves treated with dilute alkali solution and (**C**) leaves after *Trichoderma viride* fermentation; Morphology of *Eucommia ulmoides* leaves observed by SEM: (**D**) dried raw leaves, (**E**) leaves treated with dilute alkali solution and (**F**) leaves after *Trichoderma viride* fermentation.

The morphology of the samples is shown in Figure 9. The surface of the original *Eucommia ulmoides* leaves is very flat and the structure is complete (Figure 9A). The *Eucommia ulmoides* leaves extracted by surfactant gradually changed from leaves with complete structure to a state of loose structure (Figure 9B). As can be seen from Figure 9C, the structure of *Eucommia ulmoides* leaves fermented by *Trichoderma viride* was no longer complete and took on the shape of fragments. Furthermore, the results of SEM showed that the surface of the original *Eucommia ulmoides* leaves was relatively flat and had more impurities (Figure 9D). After being extracted by surfactant SDBS, the surface of *Eucommia ulmoides* leaves became rough (Figure 9E). The leaves of *Eucommia ulmoides* leaves cultured by *Trichoderma viride* showed a loose and porous structure that was beneficial to the leaching of effective components (Figure 9F). The results showed that the pretreatment of *Eucommia ulmoides* leaves by surfactant SDBS combined with *Trichoderma viride* was a favorable method for the extraction of gutta-percha.

### 3.8. XRD Analysis

**Figure 10:**
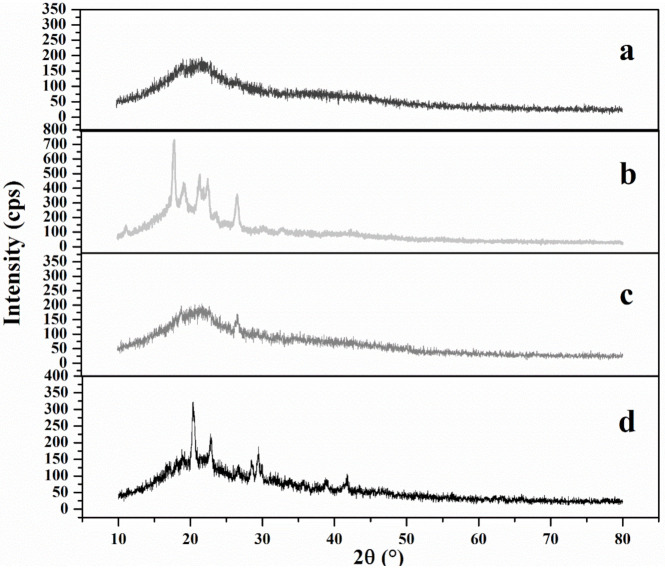
XRD detection pattern of each component after treatment. (**a**) Gutta-percha; (**b**) raw leaves; (**c**) leaves treated with dilute alkali solution; (**d**) leaves after *Trichoderma viride* fermentation.

XRD can detect the law of crystal state in the material and determine the crystallization level of the material. As shown in Figure 10a, there were two obvious peaks in the spectrum of gutta-percha (at 2θ = 19.28 and 23.12). The XRD curve of the original *Eucommia ulmoides* leaves (Figure 10b) was almost the same as that of the leaves treated with microwave ultrasound-assisted surfactant SDBS (Figure 10c), and there was no obvious characteristic peak. This shows that there was no obvious change in the crystal structure of *Eucommia ulmoides* leaves. In addition, the characteristic peaks of gutta-percha were not detected in b and c curves, which may have been due to the fact that a large amount of cellulose in the amorphous region of *Eucommia ulmoides* leaves masked gutta-percha, which made it difficult to detect gutta-percha. The XRD results of *Eucommia ulmoides* leaves treated with *Trichoderma viride* are shown in Figure 10d. The leaves of *Eucommia ulmoides* treated with *Trichoderma viride* had obvious characteristic peaks (at 2θ = 18.75) that were consistent with the characteristic peaks of gutta-percha. *Trichoderma viride* acts on the amorphous region of *Eucommia ulmoides* leaves structure, which reduces the amount of cellulose in the amorphous region so that the gutta-percha in the crystallization region is easier to be detected and has obvious characteristic peaks.

### 3.9. FTIR Analysis

**Figure 11:**
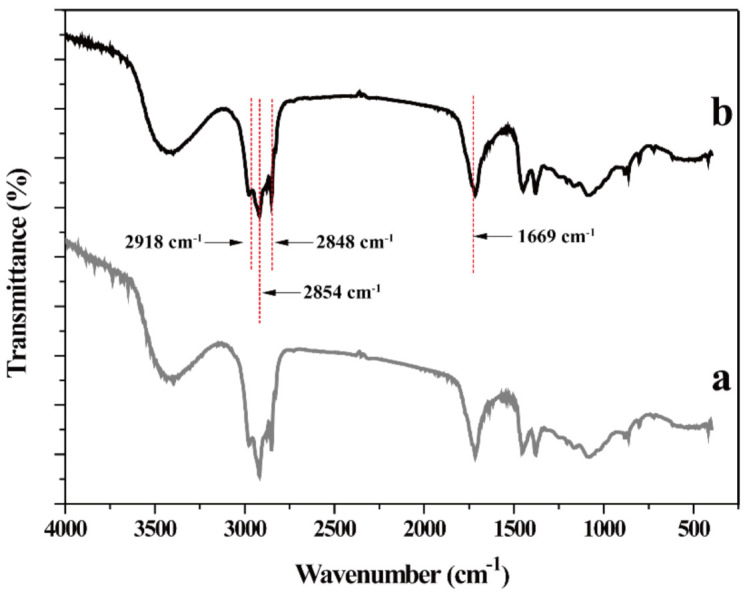
FTIR spectrum of gutta-percha. (**a**) Petroleum ether as extraction solvent; (**b**) turpentine oil as extracted solvent.

The gutta-percha extracted with conventional solvent petroleum ether was detected by FTIR spectroscopy (Figure 11a). In addition, gutta-percha extracted from turpentine as solvent was also detected by FTIR (Figure 11b). As can be seen from Figure 11b, the stretching vibration peaks of CH_3_ group appeared at 2854 cm^−1^ and 2918 cm^−1^. The stretching frequency peaks of CH_2_ and C=C appeared at 2848 cm^−1^ and 1669 cm^−1^, respectively. It was found that there was no apparent difference between two gutta-percha samples, which were isolated by petroleum ether and turpentine, respectively. This result identified that chemical structure of gutta-percha was not changed after extraction of turpentine.

### 3.10. ^1^H NMR Spectra of Gutta-Percha

**Figure 12:**
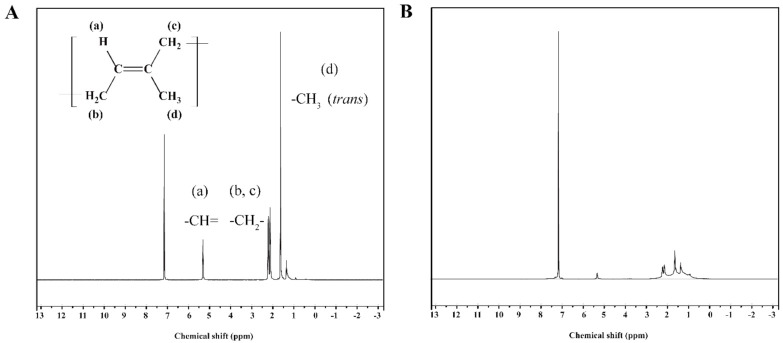
^1^H NMR spectra of gutta-percha extracted from *Eucommia ulmoides* leaves. (**A**) Petroleum ether as extraction solvent; (**B**) turpentine oil as solvent extracted.

Figure 12A shows the ^1^H NMR spectrum of gutta-percha obtained by direct extraction of *Eucommia ulmoides* leaves with petroleum ether as solvent. Figure 12B is the ^1^H NMR spectrum of gutta-percha obtained from *Eucommia ulmoides* leaves treated by surfactant SDBS and *Trichoderma viride* after turpentine extraction. The chemical shift of the signal peak (d) was about 1.60 ppm, which was the *trans*-1,4-link methyl proton peak [37]. However, the *cis*-1,4-chain of the methyl proton peak was not emerged at a chemical shift of 1.67 ppm. We thus concluded that the *cis*-1,4-polyisoprene was nonexistent in natural gutta-percha. The signals at position 2.12 ppm (c) and 2.21 ppm (b) indicated that there were two methylene proton peaks. A signal peak of olefine hydrogen atom was observed at 5.32 ppm (a). Figure 12 shows that there were almost no hydrogen signals in 1,2-chain and 3,4-chain in natural gutta-percha. Therefore, there was no 1,2-polyisoprene or 3,4-polyisoprene in natural gutta-percha. Figure 12A, B shows that the ^1^H NMR spectrum of gutta-percha extracted from petroleum ether or turpentine was the same. Turpentine can be used as a substitute solvent for petroleum ether to extract gutta-percha.

## 4. Conclusions

In this study, ultrasonic-microwave-assisted dilute alkali pretreatment was used to extract total flavonoids from *Eucommia ulmoides* leaves, and then *Trichoderma viride* fermentation and turpentine were used to extract gutta-percha to realize the cascade utilization of effective components in *Eucommia ulmoides* leaves. The extraction parameters of total flavonoids were optimized by response surface analysis. The optimum parameters were as follows: 1.5% surfactant concentration, 700 W microwave power, 30 min extraction time, and 50 mL/g liquid–solid ratio. The actual yield of total flavonoids was 1.45%. In the same way, the extraction parameters of gutta-percha were optimized by response surface method. The optimum parameters were as follows: 0.06 g/mL solid–liquid ratio, four times extraction time, 89.72 min extraction time, and 85 °C extraction temperature. The actual yield of gutta-percha was 4.38%. Gutta-percha extracted from *Eucommia ulmoides* leaves pretreated with dilute alkali solution and fungi was determined by FTIR, XRD, and ^1^H NMR. The results demonstrated that this novel method did not destroy the original structure of gutta-percha. Taken together, the extraction process not only reduces environmental pollution, but also facilitates the multi-stage utilization of resources.

## Figures and Tables

**Table 1:** Summary of reports available on production of total flavonoids extraction in *Eucommia ulmoides* leaves.

Extraction Methods	Extraction Solvent	Extraction Time (min)	Total Flavonoid Extraction Rate (%)	Reference
Ultrasound/microwave-assisted extraction	41% ethanol	26	2.454 ± 0.230	Xiang Wang et al., 2020
Refluxed	95% ethanol	360	1.39	Weixing Huang et al., 2020
Ultrasound extraction	65% ethanol	30	-	Daixiu Yuan et al., 2017
Supercritical CO_2_ extraction	80% ethanol	150	2.032	Jiaxing Li et al., 2013

**Table 2:** Experimental design matrix to screen for variables that determine the yield of total flavonoids from *Eucommia ulmoides* leaves ^a^.

No.	Central Composite Designs					No.	Central Composite Designs				
	*X*_1_	*X*_2_	*X*_3_	*X*_4_	*Y*		*X*_1_	*X*_2_	*X*_3_	*X*_4_	*Y*
1	1.25	600	25	45	0.81	16	1.75	800	35	55	1.67
2	1.75	600	25	45	1.14	17	1.0	700	30	50	0.87
3	1.25	800	25	45	0.69	18	2.0	700	30	50	1.52
4	1.75	800	25	45	1.15	19	1.5	500	30	50	1.30
5	1.25	600	35	45	1.14	20	1.5	900	30	50	0.84
6	1.75	600	35	45	1.37	21	1.5	700	20	50	1.45
7	1.25	800	35	45	0.99	22	1.5	700	40	50	1.68
8	1.75	800	35	45	1.34	23	1.5	700	30	40	1.10
9	1.25	600	25	55	1.39	24	1.5	700	30	60	1.96
10	1.75	600	25	55	1.78	25	1.5	700	30	50	1.61
11	1.25	800	25	55	1.14	26	1.5	700	30	50	1.67
12	1.75	800	25	55	1.64	27	1.5	700	30	50	1.62
13	1.25	600	35	55	1.56	28	1.5	700	30	50	1.58
14	1.75	600	35	55	1.86	29	1.5	700	30	50	1.68
15	1.25	800	35	55	1.20	30	1.5	700	30	50	1.65

^a^ The results were obtained with Design Expert 8.0.6.1 software.

**Table 3:** The results of analyses of variance to screen for variables that determine the yield of total flavonoids from *Eucommia ulmoides* leaves ^a^.

Analysis of Variance					
Source	Sum of Square	Degree of Freedom	Mean Square	*F*-Value	*p*-Value
Model ^b^	3.24	14	0.23	105.75	<0.0001 ^c^
*X*_1_	0.78	1	0.78	355.96	<0.0001 ^c^
*X*_2_	0.19	1	0.19	88.09	<0.0001 ^c^
*X*_3_	0.14	1	0.14	64.82	<0.0001 ^c^
*X*_4_	1.18	11	1.18	540.76	<0.0001 ^c^
*X*_1_ *X*_2_	0.017	1	0.017	7.95	0.0129
*X*_2_ *X*_4_	0.027	1	0.027	12.35	0.0031
*X*_3_ *X*_4_	0.031	1	0.031	14.22	0.0018
*X*_1_^2^	0.37	1	0.37	168.06	<0.0001 ^c^
*X*_2_^2^	0.60	1	0.50	273.26	<0.0001 ^c^
*X*_3_^2^	0.015	1	0.015	6.83	0.0196
*X*_4_^2^	0.028	1	0.028	12.91	0.0027
Residual	0.033	15	0.00219	-	-
Lack of fit	0.026	10	0.002551	1.74	0.2039
Pure error	0.00735	5	0.00147	-	-
Corrected total	3.28	29	-	-	-
Credibility analysis of the regression equations					
Standard deviation		Mean	Coefficient of variation		*R* ^2^
0.047		1.38	3.39		0.9852
Adjust *R*^2^		Predicted *R*^2^	Adequacy precision		
0.9761		0.9519	37.1		

^a^ The results were obtained with Design Expert 8.0 software. ^b^ *X*_1_ is the content of surfactant SDBS (%), *X*_2_ is the microwave power (W), *X*_3_ is the extraction time (min), *X*_4_ is the liquid–solid ratio (mL/g), and *Y* is the extraction yield for total flavonoids (%). ^c^ Significant at *p* < 0.05.

**Table 4:** Fermentation conditions of *Trichoderma viride*.

Variable	pH Value A	Solid–Liquid Ratio B	Inoculum Amount (%) C	Culture Time (days) D	Enzyme Activity
1	4	1:10	25	3	9.21
2	4	1:20	30	5	5.598
3	4	1:50	20	7	3.61
4	5	1:10	30	7	2.348
5	5	1:20	20	3	3.25
6	5	1:50	25	5	2.538
7	6	1:10	20	5	19.882
8	6	1:20	25	7	18.42
9	6	1:50	30	3	13.91
K1	6.1392	10.48	10.0525	8.7883	
K2	2.7083	9.0892	7.2833	9.3358	
K3	17.4025	6.6808	8.9142	8.1258	
Range	14.6942	3.7992	2.76992	1.2100	
Optimal level	A3	B1	C1	D2	
Optimum condition	6	1:10	20	5	

**Table 5:** Experimental design matrix to screen for variables that determine the yield of gutta-percha from *Eucommia ulmoides* leaves ^a^.

No.	Central Composite Designs					No.	Central Composite Designs				
	*X*_1_	*X*_2_	*X*_3_	*X*_4_	*Y*		*X*_1_	*X*_2_	*X*_3_	*X*_4_	*Y*
1	0.05	2	60	65	2.956	16	0.08	4	120	85	2.918
2	0.08	2	60	65	1.38	17	0.03	3	90	75	3.1
3	0.05	4	60	65	2.766	18	0.1	3	90	75	1.21
4	0.08	4	60	65	1.569	19	0.07	1	90	75	2.336
5	0.05	2	120	65	2.876	20	0.07	5	90	75	3.094
6	0.08	2	120	65	2.125	21	0.07	3	30	75	2.892
7	0.05	4	120	65	2.945	22	0.07	3	150	75	2.661
8	0.08	4	120	65	1.706	23	0.07	3	90	55	3.999
9	0.05	2	60	85	2.15	24	0.07	3	90	95	3.986
10	0.08	2	60	85	1.986	25	0.07	3	90	75	3.873
11	0.05	4	60	85	3.653	26	0.07	3	90	75	4.359
12	0.08	4	60	85	3.636	27	0.07	3	90	75	3.714
13	0.05	2	120	85	2.163	28	0.07	3	90	75	3.896
14	0.08	2	120	85	2.236	29	0.07	3	90	75	4.011
15	0.05	4	120	85	3.447	30	0.07	3	90	75	2.932

^a^ The results were obtained with Design Expert 8.0 software.

**Table 6:** The results of analyses of variance to screen for variables that determine the yield of gutta-percha from *Eucommia ulmoides* leaves ^a^.

Analysis of Variance					
Source	Sum of Square	Degree of Freedom	Mean Square	*F*-Value	*p*-Value
Model ^b^	18.89	14	1.35	7.52	0.0002
*X*_1_	3.51	1	3.51	19.57	0.0005
*X*_2_	1.65	1	1.65	9.17	0.0085
*X*_3_	0.00084	1	0.00084	0.004684	0.9463
*X*_4_	0.61	1	0.61	3.42	0.084
*X*_1_ *X*_4_	1.06	1	1.06	5.93	0.0278
*X*_2_ *X*_4_	1.87	1	1.87	10.42	0.0056
*X*_1_^2^	6.15	1	6.15	34.29	<0.0001 ^c^
*X*_2_^2^	3.05	1	3.05	17.01	0.0009
*X*_3_^2^	2.78	1	2.78	15.48	0.0013
*X*_4_^2^	0.0055	1	0.0055	0.031	0.8629
Residual	2.69	15	0.18	-	-
Lack of fit	1.56	10	0.16	0.69	0.7129
Pure error	1.13	5	0.23	-	-
Corrected total	21.58	29	-	-	-
Credibility analysis of the regression equations					
Standard deviation		Mean	Coefficient of variation		*R* ^2^
0.42		2.89	12.15		0.8897
Adjust *R*^2^		Predicted *R*^2^	Adequacy precision		
0.7589		0.5084	9.758		

^a^ The results were obtained with Design Expert 8.0 software. ^b^ X_1_ is the solid–liquid ratio (g/mL), X_2_ is the extraction times, X_3_ is the extraction time (min), X_4_ is the extraction temperature (°C), and Y is the extraction yield for gutta-percha (%). ^c^ Significant at *p* < 0.05.

**Table 7:** Comparison of yield of gutta-percha before and after *Eucommia ulmoides* leaves treatment.

Sample	Sample Quality (g)	Extraction Time (min)	Reaction Temperature (°C)	Extraction Times	Turpentine Volume (mL)	Yield of Gutta-Percha (%)	Amount of Solvent Required (mL/mg)
*Eucommia ulmoides* leaves (untreated)	5	60	85	1	100	0.66	2.91
*Eucommia ulmoides* leaves treated with *Trichoderma viride*	5	60	85	1	100	2.77	0.96
